# A New Model for Predicting Non-Sentinel Lymph Node Status in Chinese Sentinel Lymph Node Positive Breast Cancer Patients

**DOI:** 10.1371/journal.pone.0104117

**Published:** 2014-08-11

**Authors:** Miao Liu, Shu Wang, Lu Pan, Deqi Yang, Fei Xie, Peng Liu, Jiajia Guo, Jiaqing Zhang, Bo Zhou

**Affiliations:** Breast Disease Center, Peking University People's Hospital, Beijing, China; University of North Carolina School of Medicine, United States of America

## Abstract

**Background:**

Our goal is to validate the Memorial Sloan-Kettering Cancer Center (MSKCC) nomogram and Stanford Online Calculator (SOC) for predicting non-sentinel lymph node (NSLN) metastasis in Chinese patients, and develop a new model for better prediction of NSLN metastasis.

**Methods:**

The MSKCC nomogram and SOC were used to calculate the probability of NSLN metastasis in 120 breast cancer patients. Univariate and multivariate analyses were performed to evaluate the relationship between NSLN metastasis and clinicopathologic factors, using the medical records of the first 80 breast cancer patients. A new model predicting NSLN metastasis was developed from the 80 patients.

**Results:**

The MSKCC and SOC predicted NSLN metastasis in a series of 120 patients with an area under the receiver operating characteristic curve (AUC) of 0.688 and 0.734, respectively. For predicted probability cut-off points of 10%, the false-negative (FN) rates of MSKCC and SOC were both 4.4%, and the negative predictive value (NPV) 75.0% and 90.0%, respectively. Tumor size, Kiss-1 expression in positive SLN and size of SLN metastasis were independently associated with NSLN metastasis (p<0.05). A new model (Peking University People's Hospital, PKUPH) was developed using these three variables. The MSKCC, SOC and PKUPH predicted NSLN metastasis in the second 40 patients from the 120 patients with an AUC of 0.624, 0.679 and 0.795, respectively.

**Conclusion:**

MSKCC nomogram and SOC did not perform as well as their original researches in Chinese patients. As a new predictor, Kiss-1 expression in positive SLN correlated independently with NSLN metastasis strongly. PKUPH model achieved higher accuracy than MSKCC and SOC in predicting NSLN metastasis in Chinese patients.

## Introduction

Sentinel lymph node biopsy (SLNB) has taken the place of axillary lymph node dissection (ALND) in early stage breast carcinoma and its benefits in terms of morbidity have been well established [1]. But for breast cancer patients with sentinel lymph node (SLN) metastases, the benefit of ALND on survival is debated. The American College of Surgeons Oncology Group (ACOSOG) Z0011 trial showed that women with T1 and T2 tumors who undergo lumpectomy derive little additional benefit from ALND since any residual disease in the level I and II nodes appear to be effectively eradicated by postoperative irradiation and chemotherapy [2]. While in most centers, the current standard of treatment remains ALND [3]. However, 40% to 60% of patients have no disease in axillary lymph nodes other than the SLN itself and this means these patients undergo unnecessary ALND [4]–[6]. Therefore, the ability of a diagnostic test to predict the chance of NSLN involvement is critical to avoid unnecessary ALND.

In the past 10 years, multiple models have been proposed to aid in the stratification of a patient's risk of having NSLN metastasis [7]–[10], in which the nomogram developed at the Memorial Sloan-Kettering Cancer Center [11] and the model of Stanford Online Calculator (SOC) [12] are the most well-validated and widely used. However, both models were established based on a Western population. It is well known that there are some difference in the clinical characteristics and treatment modes between Chinese breast cancers patients and Westerners. Therefore, there is a clear need of studies to evaluate these nomograms in Chinese breast cancer patients.

To date, all the models predicting NSLN status are based on the routine histopathological variables of the primary tumor and its metastasis [13], [14]. It is uncertain if there are some additional biological markers that may be able to predict the risk of NSLN metastasis.

In the current study, we validated MSKCC and SOC models in predicting NSLN metastasis in Chinese breast cancer patients. In addition, we identified new factors that may predict NSLN metastasis and developed a novel predictive model specifically for Chinese patient population.

## Materials and Methods

### Study patients

We reviewed 120 consecutive, clinically lymph node negative breast cancer patients with positive SLN(s) who underwent completion ALND from January 2009 through December 2012 at the Breast Disease Center, Peking University People's Hospital (PKUPH). Approval from PKUPH's review board was obtained before data collection. Written consent has been obtained from all the patients.

### Surgery and SLN pathological evaluation

SLNs were identified using fluorescence and/or blue dye according to surgeon preference. Intraoperative frozen section was performed on all SLNs. The SLN was cut longitudinally into 2 halves. Half of the node was frozen for immediate examination, and up to 2 sections were stained with hematoxylin and eosin (H&E). The other half node was fixed in formalin and embedded in paraffin, and up to 2 sections were stained with H&E. Immunohistochemical stain was not routinely used in the diagnosis of SLN metastasis.

The size of metastatic tumor deposits within all nodes was categorized according to the American Joint Committee on Cancer Staging Manual (7th edition) as follows: isolated tumor cells (ITC) were defined as tumor deposit of 0.2 mm or less (pN0i+), micrometastases (MI) were defined as tumor deposit of more than 0.2 mm but not more than 2 mm (pN1mi), and macrometastases were defined as tumor deposit of more than 2 mm (pN1).

Axillary dissection was performed if SLN was positive by frozen section analysis. Patients with SLN metastases who were not detected during operation generally underwent completion ALND at a later date. For all additional nodes identified by completion ALND, routine H&E analysis was conducted on a single section of each node.

### MSKCC nomogram and SOC

The MSKCC nomogram is based on 9 histopathologic variables: primary tumor size (in centimeters), primary tumor type (ductal/lobular) and nuclear grade (1–3), number of positive SLNs, number of negative SLNs, method of SLN detection (frozen, routine H&E, serial H&E, or immunohistochemistr [IHC]), lymphovascular invasion (LVI) (yes/no), multifocality (yes/no) and estrogen receptor positivity (yes/no).

The Stanford nomogram requires 3 histopathologic variables: primary tumor size (in centimeters), size of SLN metastasis (ITC, MI, and macrometastases) and LVI (yes/no).

### Clinical and pathological characters

Clinical and pathological data collected for each case included age, tumor size (pathological size of the invasive carcinoma), tumor site (superior internal, inferior internal, superior external, inferior external and central quardrant), tumor type (ductal or lobular carcinoma, ductal and lobular mixed carcinoma, special type carcinoma (such as mucinous adenocarinoma and so on), nuclear grade, multifocality, presence of LVI, molecular subtype, expression of estrogen receptor (ER), progesterone receptor (PR), Her-2, Ki67, CK5/6, p53, EGFR, E-cadherin, Kiss-1, nm23 in tumor, expression of Kiss-1 and nm23 in positive SLNs, reduction of Kiss-1 expression from tumor to positive SLN (Kiss-1 positive in breast tumor while negative in positive SLN), reduction of nm23 expression from tumor to positive SLN (nm23 positive in breast tumor while negative in positive SLN), method of detection of SLN metastases (frozen-section analysis [frozen], routine H&E [routine H&E], H&E stains of serial sections [serial HE]), number of positive SLN, number of negative SLN, number of total SLN, ratio between positive SLNs and total amount of SLN, size of SLN metastasis (ITC, MI, and macrometastases), and extracapsular invasion (ECI) at positive SLNs, ratio of SLN metastasis size and positive SLN size with total of 30 factors.

### Statistical analysis

We used the 120 patients' data to validate the performance of MSKCC and SOC nomogram. Model discrimination was assessed by calculating the area under the receiver operating characteristic curve (AUC) with 95% confidence interval (95% CI). The nomograms were further assessed the ability to accurately identify patients at very low risk for NSLN metastasis using false-negative rate (FN), negative predictive value (NPV), sensitivity, specificity and overall predictive accuracy with the cutoff value of 10%.

We tried to develop a novel predictive model specifically for Chinese patients according to the records of the first 80 consecutive patients from the 120 patients. First, univariate analysis of the 30 factors described above was performed to determine which one was associated with NSLN metastases. Chi-squared test and Fisher's exact test were used for categorical variables and Mann–Whitney *U* test for continuous variables. The predictors with *p* value less than 0.05 were included in the multivariate analysis. All variables with *p* value<0.05 were considered statistically significant in the multivariate analysis and were then included into a logistic regression analysis using backward stepwise method to create the final predictive model.

The resulting multivariate predictive model was then validated with the 80 series themselves. Discrimination of the model was assessed using AUC and the Hosmer–Lemeshow goodness-of-fit test. AUC, false-negative rate, negative predictive value, sensitivity, specificity and overall predictive accuracy with the cutoff value of 10% were compared between the new model, MSKCC model and SOC model using the sample of additional 40 patients from the 120 patients.

SPSS16.0 software was used for statistical analyses.

## Results

**Figure 1:**
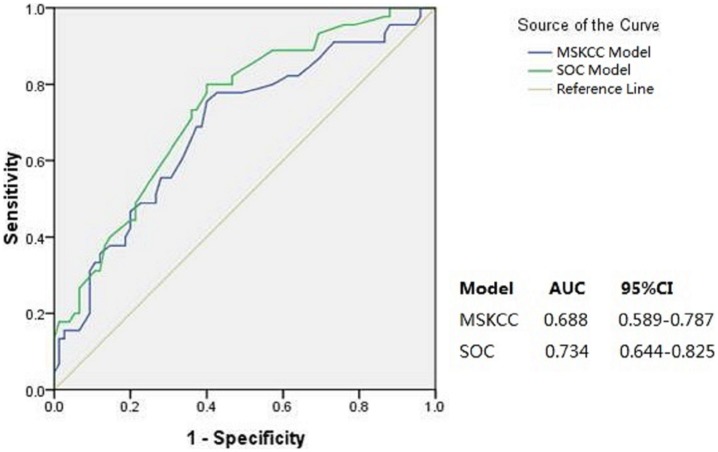
Area under the receiver operating characteristic curve (AUC) for MSKCC and SOC models(n = 120). Diagonal line represents an AUC of 0.5, indicating a score equal to chance.

Forty five (37.5%) of the 120 patients from PKUPH had NSLN metastasis and the descriptive tumor and nodal characteristics of this cohort used in the MSKCC and Stanford nomograms are listed in Table 1. Compared with the MSKCC [11] and Stanford studies [12], difference was found in age, tumor type, nuclear grade, LVI, multifocality, method of SLN detection and size of positive SLN metastasis. The ROC curves generated by MSKCC and Stanford nomograms are shown in Figure 1. The AUC value of the Stanford and MSKCC models was 0.734(95% CI, 0.644–0.825) and 0.688(95% CI, 0.589–0.787), respectively.

**Table 1:** Clinical data of patients with positive SLNs from MSKCC, SOC and PKUPH.

	MSKCC		SOC			PKUPH		
Total patients	702		285			120		
Patient and Tumor Characteristics	n	%	n	%	Mean	n	%	Mean
Age(years)					55.8			52.53
≤50	290	41.3				59	49.2	
>50	412	58.7				61	50.8	
Tumor size (cm)								
≤1.0	155	22.1	31	10.9		25	20.8	
>1.0 to ≤2.0	312	44.4	125	43.9		64	53.3	
>2.0 to ≤3.0	154	21.9	129	45.2		22	18.3	
>3.0	81	11.6				9	7.5	
Tumor type								
Ductal	618	88.0	246	86.3		73	60.8	
Lobular	84	12.0	27	9.5		6	0.1	
Mixed			11	3.9		38	31.7	
Special type			1	0.4		3	2.5	
Nuclear grade								
I	22	3.1	91	31.9		13	10.8	
II	321	45.7	120	42.1		74	61.7	
III	275	39.2	74	26.0		27	22.5	
Lobular	84	12.0				6	5.0	
LVI								
Yes	284	40.5	95	33.3		14	11.7	
No	418	59.5	118	41.4		106	88.3	
Unknown			72	25.3				
Multifocality								
Yes	197	28.1				9	7.5	
No	505	71.9				111	92.5	
ER status								
+	567	80.8	194	68.1		101	84.2	
−	135	19.2	38	13.3		19	15.8	
Unknown			53	18.6				
Method of SLN detection								
Frozen section	463	66.0				99	82.5	
Routine HE	65	9.3	220	77.2		21	17.5	
Serial HE	78	11.1						
IHC	63	9.0	65	22.8				
Unknown	33	4.7						
Number of positive SLNs								
1	488	69.5	208	73.0		74	61.7	
2	161	22.9	61	21.4		30	25.0	
>2	53	7.6	16	5.6		16	13.3	
Number of negative SLNs								
0	271	38.6				36	30.0	
1	183	26.1				17	14.2	
2	102	14.5				20	16.7	
>2	146	20.8				47	39.2	
Size of SLN metastasis (mm)								
≤2			264	92.7		44	36.7	
>2			21	7.4		76	63.3	
NSLN positive								
+	264	37.6	101	35.4		45	37.5	
−	438	62.4	184	64.6		75	62.5	

When patients with a low risk of having NSLN disease were examined, we found that, based on the MSKCC nomogram, 8 patients in this study cohort had a 10% predicted probability of having NSLN metastasis. Of these 8 patients, 2 had positive NSLNS with a FN rate of 4.4% and NPV of 75.0%. The sensitivity and specificity was 95.6% and 8.0%, respectively, with an overall predictive accuracy of 40.8%. Compared with MSKCC nomogram, 20 patients by the SOC model were predicted to have a 10% probability of having NSLN metastasis, of whom 2 had positive NSLNS with a FN rate of 4.4% and NPV of 90.0%. The sensitivity and specificity was 95.6% and 24.0%, respectively, with an overall predictive accuracy of 50.8% (Table 2).

**Table 2:** MSKCC and SOC Models at 10% Predicted Probability Cut-off Values Applied to PKUPH Data (n = 120).

Predicted Probability of NSLN Metastasis	Modle	Clinical utility n(%)	FN n(%)	NPV (%)	Sensitivity (%)	Specificity (%)	Overall predictive accuracy (%)
≤10	MSKCC	8(6.7)	2(4.4)	75.0	95.6	8.0	40.8
	SOC	20(16.7)	2(4.4)	90.0	95.6	24.0	50.8

Univariate analysis of the patients with additional metastases on NSLN and those with no additional metastases on NSLN are given in Table 3. Significant difference was found between the two groups in the tumor size, tumor type, LVI, tumor p53 expression, expression of kiss-1 in positive SLN, decrease of kiss-1 expression from tumor to positive SLN, number of positive SLN, size of SLN metastasis, the ratio of SLN metastasis size and positive SLN. Expression of Kiss-1 in positive SLN is correlated with decrease of Kiss-1 expression from tumor to positive SLN. Size of SLN metastasis is correlated with the ratio of SLN metastasis size and positive SLN size. To avoid the interaction of these predictors, decrease of Kiss-1 expression from tumor to positive SLN and the ratio of SLN metastasis size with positive SLN size were not included in the multivariate analysis.

**Table 3:** Univariate analyses for patient and tumor and SLN characteristics associated with NSLN metastases (n = 80).

variable	characteristic	NSLN(+)		NSLN (−)		M(Q25,Q75)*	*p*
		n = 27		n = 53			
		n	%	n	%		
X1	Age (year)						0.10
	≤50	16	59.3	21	39.6		
	>50	11	40.7	32	60.4		
X2	Tumor site						0.79
	Superior external	12	44.4	26	49.1		
	Inferior external	6	22.2	8	15.1		
	Superior internal	8	29.6	15	28.3		
	Inferior internal	1	3.7	4	7.5		
X3	Tumor size(cm)						0.01
	≤1.0	3	11.1	15	28.3		
	>1.0 to ≤2.0	14	51.9	27	50.9		
	>2.0 to ≤3.0	4	14.8	10	18.9		
	>3.0	6	22.2	1	1.9		
X4	Tumor type						0.04
	Ductal	15	55.6	40	75.5		
	Lobular	5	18.5	1	1.9		
	Mixed	7	25.9	11	20.8		
	Special type	0	0	1	1.9		
X5	Nuclear grade*						0.74
	I	1	3.7	3	5.7		
	II	16	59.3	33	62.3		
	III	5	18.5	16	30.1		
	Lobular	5	18.5	1	1.9		
X6	LVI						0.005
	Yes	6	22.2	1	1.9		
	No	21	77.8	52	98.1		
X7	Multifocality						0.22
	Yes	4	14.8	3	5.7		
	No	23	85.2	50	94.3		
X8	ER status						>0.99
	+	22	81.5	44	83		
	−	5	18.5	9	17		
X9	PR status						0.28
	+	21	77.8	35	66		
	−	6	22.2	18	34		
X10	Her-2 expression						0.29
	+	5	18.5	5	9.4		
	−	22	81.5	48	90.6		
X11	Ki67						0.93
	<14%	15	55.6	30	56.6		
	≥14%	12	44.4	23	43.4		
X12	Molecular subtype						0.80
	Luminal A	11	40.7	22	41.5		
	Luminal B	11	40.7	22	41.5		
	Her-2(+)	3	11.1	3	5.7		
	Triple negative	2	7.4	6	11.3		
X13	CK5/6						0.32
	+	2	7.4	9	17		
	−	25	92.6	44	83		
X14	EGFR						0.60
	+	7	25.9	11	20.8		
	−	20	74.1	42	79.2		
X15	P53						0.02
	+	14	51.9	14	26.4		
	−	13	48.1	39	73.6		
X16	E-cad						0.38
	+	19	70.4	42	79.2		
	−	8	29.6	11	20.8		
X17	nm23(T)						0.53
	+	24	88.9	43	81.1		
	−	3	11.1	10	18.9		
X18	nm23(SLN)						0.68
	+	12	44.4	21	39.6		
	−	15	55.6	32	60.4		
X19	nm23↓						0.69
	Yes	14	51.9	25	47.2		
	No	13	48.1	28	52.8		
X20	Kiss-1(T)						0.20
	+	25	92.6	42	79.2		
	−	2	7.4	11	20.8		
X21	Kiss-1(SLN)						0.01
	+	7	25.9	31	58.5		
	−	20	74.1	22	41.5		
X22	Kiss-1↓						0.001
	Yes	20	74.1	19	35.8		
	No	7	25.9	34	64.2		
X23	SLN Detection						>0.99
	Frozen section	23	85.2	44	83		
	HE	4	14.8	9	17		
X24	No. of positive SLNs						0.004
	1	10	37	40	75.5		
	2	10	37	8	15.1		
	>2	7	25.9	5	9.4		
X25	No. of negative SLNs						0.29
	0	11	40.7	15	28.3		
	1	1	3.8	8	15.1		
	2	6	22.2	8	15.1		
	>2	9	33.3	22	41.5		
X26	No. of SLNs						0.64
	1	4	14.8	12	22.6		
	2	4	14.8	9	17		
	>2	19	70.4	32	60.4		
X27	Positive SLNs/SLNs					0.5 (0.3, 1.0)	0.11
X28	ECI at positive SLNs						0.26
	Yes	26	96.3	46	86.8		
	No	1	3.7	7	13.2		
X29	Size of SLN metastasis Jo (mm)						0.000
	≤2	2	7.4	28	52.8		
	>2	25	92.6	25	47.2		
X30	SLN metastasis size/淋巴结大小					0.5(0.14,0.91)	0.000
	positive SLN size						

*M:Median, Q25:25% of Quartile, Q75:75% of Quartile.

**Figure 2:**
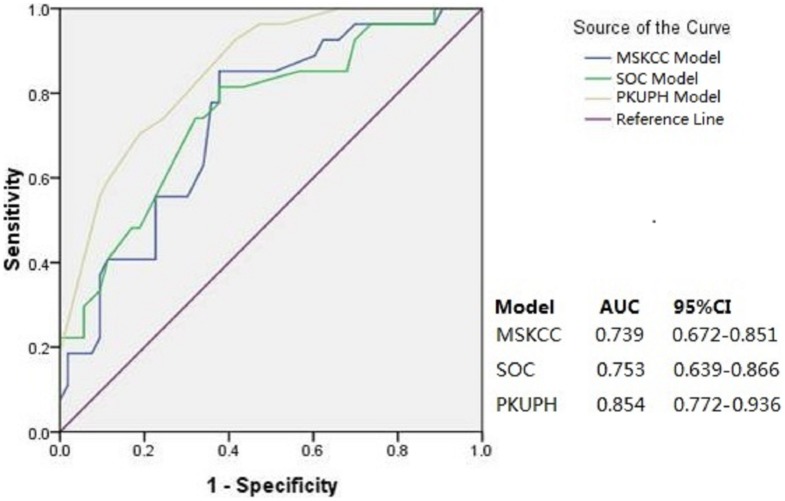
Area under the receiver operating characteristic curve (AUC) for MSKCC, SOC, and PKUPH models (n = 80).

By multivariate analysis, tumor size, expression of Kiss-1 in positive SLN and the size of SLN metastasis remained significantly predictive of NSLN status (p<0.05). The results of the backward stepwise binary logistic regression analysis are given in Table 4. The multivariate logistic regression analysis produced the following mathematical predictive model (PKUPH model) for NSLN status in our patient cohort, with the Y′ denoting the probability of NSLN metastases:
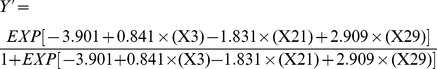
Goodness-of-fit of the model was assessed by Hosmer-Lemeshow (HL) test that produced a *p* value of 0.967, indicating that the multivariate model fits well for the patient population. This new model's discrimination was compared with the MSKCC and SOC models using the 80 patients themselves. The AUC value of the PKUPH model was 0.854 (95% CI, 0.772–0.936) while the AUC value of the MSKCC and Stanford model was 0.739 (95% CI, 0.627–0.851) and 0.753 (95% CI, 0.639–0.866), respectively(Figure 2).

**Table 4:** Multivariable logistic regression of clinicopathologic data and NSLN involvement (n = 80).

Factor	Characteristic	*β*	*S.E*	*p*	*OR*	95%*CI*
X3	Tumor size	0.904	0.449	0.044	2.47	1.024–5.952
X4	Tumor typr	0.363	0.404	0.368	1.44	0.652–3.171
X6	LVI	1.280	1.361	0.347	3.60	0.249–51.833
X15	p53	1.289	0.719	0.073	3.63	0.887–14.860
X21	Kiss-1(SLN)	−1.791	0.686	0.009	0.17	0.043–0.641
X24	No. of positive SLNs	0.409	0.411	0.321	1.50	0.672–3.370
X29	Size of SLN metastasis	2.371	0.895	0.008	10.71	1.853–61.907
	Constant	−5.469	1.629	0.001	0.004	

*S.E*: Standard error.

*OR:* Odds ratio.

CI: Confidence intervals.

**Figure 3:**
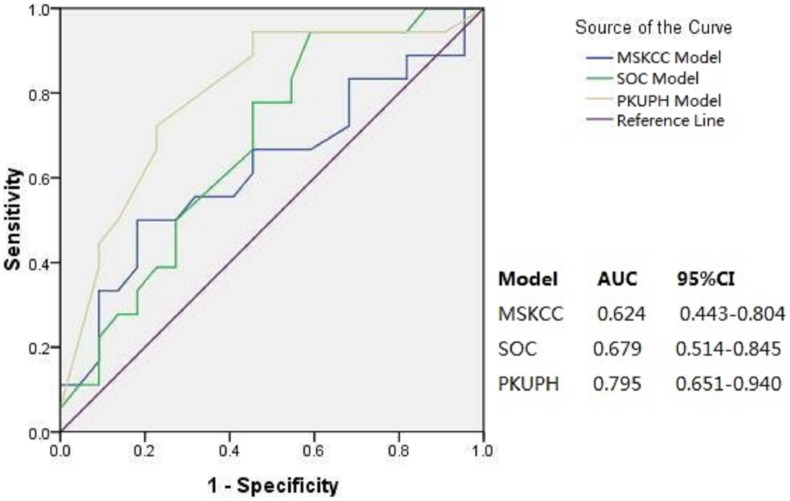
Area under the receiver operating characteristic curve (AUC) for MSKCC, SOC, and PKUPH models (n = 40).

The new model was further validated using the additional 40 patients. The AUC value of the PKUPH model was 0.795 (95% CI, 0.651–0.940) that was superior to the AUCs of 0.624 (95% CI, 0.443–0.804) and 0.679(95% CI, 0.514–0.845), generated by MSKCC and Stanford nomograms, respectively (Figure 3). The ability of these three models predicting patients with a low probability of non-SLN metastases was further compared with a cutoff value of 10%. The PKUPH model had a FN rate of 5.6%, NPV of 92.3%, specificity of 54.5%, sensitivity of 94.4%, and overall predictive accuracy of 72.5%. The FN rate, NPV, specificity, sensitivity and overall predictive accuracy of MSKCC model were 11.1%, 50%, 9.1%, 88.9% and 45%, respectively. The FN rate, NPV, specificity, sensitivity and overall predictive accuracy produced by SOC model was 5.6%, 80%, 18.2%, 94.4% and 52.5% (Table 5).

**Table 5:** MSKCC, SOC and PKUPH Models at 10% Predicted Probability Cutoff Values Applied to PKUPH Data (n = 40).

Predicted Probability of NSLN Metastasis	Model	Clinical utility n(%)	FN n(%)	NPV (%)	Sensitivity (%)	Specificity (%)	Overall predictive accuracy (%)
≤10	MSKCC	4(10.0)	2(11.1)	50.0	88.9	9.1	45.0
	SOC	5(12.5)	1(11.1)	80.0	99.4	18.2	52.5
	PKUPH	13(32.5)	1(5.6)	92.3	99.4	54.5	72.5

## Discussion

With the adoption of SLN biopsy, a new clinical conundrum has become commonplace: should a completion ALND be performed for a patient with a positive SLN biopsy? It is difficult to accurately estimate the risk of NSLN metastases for an individual breast cancer patient using the present methods, so ALND has been considered the golden standard for breast cancer patients with SLN metastasis.

Some tumor and nodal characteristics have been identified as risk factors for the presence of NSLN metastasis [13], [15]–[17]. The presence of predictive model provides a good way simultaneously including several variables that are acquired through mathematical method in a large population. The MSKCC model was the first nomogram to predict NSLN metastasis following a positive SLN biopsy [11], which produced an AUC of 0.72 versus the clinicians' AUC of 0.54 [18]. Subsequent validation studies have shown an AUC ranging from 0.58 to 0.78 in other centers [19]–[21]. In 2008, a modified nomogram using only 3 histopathologic variables was developed at Stanford [12]. The SOC has demonstrated an AUC of 0.64 to 0.73 by validation that seems to perform better than the MSKCC nomogram [22]–[24].

Predictive models are known to work best in the centers where they were developed and therefore require validation in other independent patient populations before being adapted to clinical use. We validated these two models in 120 Chinese breast cancer patients. Our result showed that the AUC generated by MSKCC and SOC nomogram were 0.688 and 0.734, respectively, which are inferior to their original AUC values(0.77 and 0.74). A NSLN predictive model is to select patients at low risk with NSLN involved mainly, but both MSKCC and SOC models showed poor clinical utility in Chinese breast cancer patients by selecting only 8 (6.7%) and 20 (16.7%) patients when a 10% cut-off value was used. The specificity of these two models was only 8.0% and 24%, respectively, which was inferior to most other validation studies in Western cancer centers.

The different validation results between our center and Western ones might be due to the different tumor characteristics between Chinese and Western countries population (Table 1). In China, the number of pre-menopausal breast cancer patients is usually larger than that of Western countries [25]. For MSKCC nomogram, tumor type is a predictive factor only including ductal and lobular tumor two types while there are 31.7% patients having ductal and lobular mixed tumors in our study population. LVI has been found to be correlated strongly with NSLN positivity by some authors [11], [12]. So both MSKCC and SOC models include LVI as a predictor. While the rate of LVI is 11.7% in our patient population which is much lower than the 40.5% in the MSKCC patients and 33.3% in SOC patients. For the diagnosis method of SLN metastasis, serial H&E and IHC have been widely used in Western cancer centers, while these are rarely used in China. It is clear that IHC is more sensitive than H&E in detecting micrometastases and that routine H&E analysis is more sensitive than frozen-section analysis. There is a correlation between method of detection and volume of tumor [11] and it has also been indicated that method of detection was correlated with the size of the SLN metastasis [26], [27]. We found the number of ITC and micrometastasis in our 120 patients is 0% and 36.7%, respectively, which is much lower than those in Stanford and other studies. Given the dramatic difference exists between Chinese patients and Westerners, a predictive model based on Chinese population is in great need.

Some histopathological variables of the primary tumor and its metastasis have been identified to correlate with the NSLN status. While, the estimates of risk for any given characteristic vary considerably among studies. Up to now, the size for primary tumor, grade of primary tumor, the maximum size of positive SLNs, LVI, extracapsular invasion in SLN, ER, PR and HER-2 status were the mostly analyzed risk factors in other studies. In addition to these factors, we collected biomarkers as Ki67, CK5/6, p53, EGFR, E-cadherin, Kiss-1, nm23 to analyze their relationship with NSLN involvement. By univariate analyses, we found that 9 variables were correlated to NSLN metastasis: tumor size, tumor type, LVI, expression of p53 in tumor, expression of Kiss-1 in positive SLNs, decrease of kiss-1 expression from tumor to positive SLN, number of positive SLN, size of SLN metastasis, and the ratio of SLN metastasis size and positive SLN size. In the multivariable logistic regression analysis, tumor size, expression of kiss-1 in positive SLN and the size of SLN metastasis were each associated with the likelihood of NSLN metastases. We adopted these three factors to develop our new model to predict the probability of NSLN metastasis.

Tumor size is an important factor influencing the lymph node involvement in breast cancer. Increasing tumor diameter has been shown to increase the overall risk of metastatic axillary lymph nodes and therefore the likelihood of tumor-positive SLNB [28]. It seems reasonable that risk factors for axillary lymph node metastases in general turn out to be risk factors for nonsentinel metastases after tumor-positive SLNB. This fact has been confirmed in some studies. Hwang et al reported no NSLN metastases in patients with T1a lesions whereas patients with T2, T3 and T4 tumors were associated with positive NSLNs in 54%, 77% and 80% of cases, respectively [13]. Similar data were presented by Kamath [26], Joseph [16] and Chu [29] that a NSLN metastasis rate of 13% for T1b lesions, 38% for T2, and 71% for T3 tumors. Both MSKCC and SOC models include tumor size as a predictive factor. In the current study, tumor size was found to be an independent risk factor for NSLN metastasis through univariate and multivariate analyses. Therefore we included tumor size in our new model.

The size of SLN involvement has been identified as a significant predictor of NSLN metastases. Weiser et al. reported that patients with a metastasis size ≤2 mm in SLN have very low risk of NSLN metastasis [30]. Chu et al. [29] examined the SLNs and NSLNs of 194 patients and found that 47% of the patients having positive SLNs with macrometastasis (≥2 mm) could be identified NSLN metastasis only through H&E method. Consistently, other studies found that SLN metastasis size ≥2 mm was an independent predictor of NSLN metastasis [5],[13],[31]. Kohrt et al. [12] used the size of SLN metastasis as one of the three factors in the SOC model and many studies have validated this model with good results. While the MSKCC model didn't include the size of SLN metastasis. The authors did pointed out that the absence of size determination for the SLN metastases was a limitation of the MSKCC model [11]. In our study, we proved the size of SLN metastasis remained significantly predictive of NSLN status and adopted this variable in our new model.

The current study is the first study to analyze the impact of tumor and positive SLN Kiss-1 expression for NSLN involvement. Kiss-1 has been identified as a putative human metastasis suppressor gene in melanomas [32]. It has also been suggested as a potential metastasis suppressor in breast cancer cells without affecting tumorigenicity [33]. Mitchell and colleagues [34] found the loss of Kiss-1 gene expression in highly metastatic breast cancer cell lines. Stark et al [35] determined the expression of Kiss-1 in primary breast tumor and brain metastatic foci, and found that Kiss-1 expression in brain metastatic tumor was 10 times lower than that in breast tumor, indicating that Kiss-1 gene might be involved in breast cancer metastasis. Kostadima et al [36] also have reported that the positive rate of Kiss-1 is only 3% in lymph node positive breast cancer, supporting the anti-metastatic role of the Kiss-1 for breast cancer. Our previous study has indicated that Kiss-1 is the most important and independent impact factor for lymph node metastases in breast cancer [37]. In the current study, we found that patients with a reduction of Kiss-1 expression in the positive SLN compared with the primary tumor have a higher chance for positive NSLN (p<0.05). Interestingly, primary breast cancer Kiss-1 expression was not correlated with NSLN metastasis. While Kiss-1 positive in metastatic SLN was a strong negative predictive factor of NSLN involvement. Therefore, we have included the SLN Kiss-1 expression in our predictive model.

A good breast cancer NSLN metastasis predictive model depends on not only its predictive ability but also its clinical utility convenience. To our knowledge, to date, the majority of models with a low number of variables have performed inferiorly to models with more variables. While it is interesting that the SOC model with 3 variables is comparable to the MSKCC nomogram including 9 variables. Thus, factors with strong predictive strength may be more important than the number of variables for a model. Our new model has 3 variables among which the tumor size and the size of SLN metastasis are the same ones as SOC model. While, we have chosen Kiss-1 in positive SLN instead of LVI for our model. As compared with MSKCC and SOC models, our new model performed better on AUC and ability to select patients at low risk of NSLN involvement at least in our center.

There are some limitations in our model. First, the validation series of this study was rather small and from a single center. IN addition, Kiss-1 expression in positive SLN is not a routine pathological test. Further multicenter studies are warranted to validate our new model.
